# Nanofat in Plastic Reconstructive, Regenerative, and Aesthetic Surgery: A Review of Advancements in Face-Focused Applications

**DOI:** 10.3390/jcm12134351

**Published:** 2023-06-28

**Authors:** Simone La Padula, Martina Ponzo, Mariagiovanna Lombardi, Vincenzo Iazzetta, Concetta Errico, Gianmarco Polverino, Francesca Russo, Luca D’Andrea, Barbara Hersant, Jean Paul Meningaud, Giovanni Salzano, Rosita Pensato

**Affiliations:** 1 Department of Plastic and Reconstructive Surgery, Federico II University of Naples, Via Pansini 5, 80131 Naples, Italy; dottoressamartinaponzo@gmail.com (M.P.); lmariagiovanna@gmail.com (M.L.); iazzetta.vin@gmail.com (V.I.); concettaerrico@me.com (C.E.); gianmarcopolverino94@gmail.com (G.P.); francescarusso318@gmail.com (F.R.); dandrea.luca91@gmail.com (L.D.); rositapensato@gmail.com (R.P.); 2 Department of Plastic, Reconstructive and Maxillo-Facial Surgery, Henri Mondor Hospital, University Paris, XII, 51 Avenue du Maréchal de Lattre de Tassigny, 94000 Créteil, France; barbara.hersant@gmail.com (B.H.); meningaud@me.com (J.P.M.); 3 Maxillofacial Surgery Unit, Federico II University of Naples, 80131 Naples, Italy; giovannisalzanomd@gmail.com

**Keywords:** nanofat, stromal vascular tissue, regenerative medicine, fat graft, facial rejuvenation, lipomodelling, a review

## Abstract

Nanofat is a relatively novel technique in fat grafting that has gained significant interest in the fields of regenerative medicine, aesthetic and translational research. It involves the extraction of autologous fat from a patient, which is then transformed into “nanofat”, consisting of small fat particles with a diameter of less than 0.1 mm and containing high concentrations of stem cells and growth factors. This article focuses on the use of nanofat in facial rejuvenation and its potential for lipomodelling. Fat tissue is a “stem cell depot” and nanofat contains many stem cells that can differentiate into various cell types. The Lipogem technology, developed in 2013, enables the isolation of nanofat with an intact perivascular structure, utilizing the high concentration of mesenchymal stromal cells near the pericytes of the adipose vascular system. Nowadays nanofat is used primarily for cosmetic purposes particularly in rejuvenating and improving the appearance of the skin, especially the face. Indeed, it has wide applicability; it can be used to treat fine lines, wrinkles, acne scars, sun-damaged skin, scar repair, and as an alopecia treatment. However, further studies are needed to assess the long-term efficacy and safety of this technique. In conclusion, nanofat is a safe and minimally invasive option for tissue regeneration with considerable therapeutic potential. This study reviews the application and effects of nanofat in regenerative medicine and facial cosmetic surgery.

## 1. Introduction

**Figure 1:**
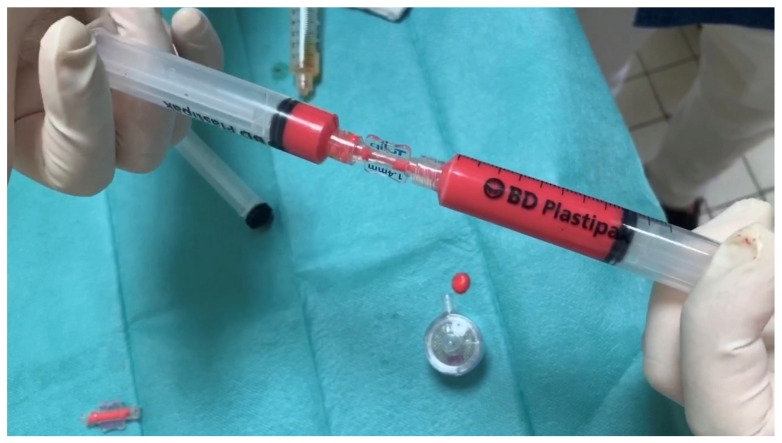
This picture shows how adipose tissue is mechanically emulsified to obtain nanofat by the authors’ method.

Nanofat is an emerging technique for fat grafting that is gaining popularity in the fields of regenerative medicine, aesthetics, and translational research. The power of mesenchymal stromal cells (MSC) has been known since the 1970s and recent studies have shown that MSCs are present in multiple tissues such as adipose tissue. Indeed, adipose tissue is a major reservoir of MSCs and accessible via liposuction techniques daily used in plastic surgery [1]. Hence it has become increasingly evident that MSCs used by adipose tissue transfer have a considerable interest for regenerative surgery. This treatment became known as fat grafting, lipofilling, and lipomodelling. Rigotti et al. in 2007 showed for the first time how the injection of adipose tissue in severe radiation lesions improved tissue hydration and neo-angiogenesis. In fact, by producing appropriate support MSCs promoted neo-angiogenesis and reduced tissue inflammation. Capitalizing on the properties of MSCs, Magalon et al. used this treatment to cure scleroderma. On a parallel track, Yoshimura et al. used fat enriched with SVF for breast augmentation with good results. A new era began when Tonnard et al. in 2013 described a mechanical digestion of adipose tissue, a simple process termed “nanofat grafting”. This technique is based on the use of autologous fat and represents a new concept in the field of lipofilling. In this technique, fat is harvested from the patient and transformed into nanofat, which is composed of small fat particles (less than 0.1 mm in diameter) containing a high concentration of stem cells and growth factors (Figure 1) [2]. This article aims to review the use of nanofat specifically in facial rejuvenation and to address new developments and concerns in this area since its introduction in 2013. Nanofat grafting has a wide range of clinical applications, including scar repair, alopecia treatment, breast tissue regeneration, and tissue regeneration in general. This technique is particularly useful for lipomodelling as it contains many stem cells that can differentiate into various cell types, such as adipocytes, osteoblasts, and chondrocytes [3]. Fat is a well-organized and compact tissue that contains a heterogeneous population of cells and is often referred to as a “stem cell depot” due to its abundance of stem cells [3]. Researchers typically identify nanofat using electron microscopy, either transmission electron microscopy (TEM) or scanning electron microscopy (SEM). The first method involves passing a beam of electrons through an ultra-thin sample of nanofat and provides high-resolution images that can reveal the internal structure of the nanofat particles. The second involves scanning a focused beam of electrons across the surface of a sample. As the electrons interact with the sample, various signals are generated, and these signals are then used to create a detailed 3D image of the surface topography of the nanofat particles. TEM allows researchers to observe the size, shape, and arrangement of lipid droplets, cell fragments, and other components within nanofat. SEM is particularly useful for examining the surface morphology and texture of nanofat. By employing electron microscopy techniques, researchers can gain insights into the ultrastructure and composition of nanofat, allowing for a better understanding of its properties and potential mechanisms of action. This information can help optimize the preparation and application of nanofat in various medical and cosmetic procedures. Nanofat is composed of vascular stromal cells, including mesenchymal stromal cells (MSCs), along with a mixture of lipids, growth factors, and cytokines, with a high expression of CD34 and CD49d [1,2,3,4,5,6,7,8,9,10,11,12,13,14,15,16,17,18,19]. It has been established that nanofat contains adipose-derived stem/stromal cells (ASCs), and its potential application in regenerative medicine has been the subject of numerous studies [19]. The Lipogem technology, developed in 2013 by Lipogems International SpA (Milan, Italy), enables the isolation of nanofat with an intact perivascular structure; this technology has shown promise in regenerative medicine and has demonstrated considerable therapeutic potential in preliminary results [20,21]. However, further studies are needed to assess the long-term efficacy and safety of nanofat grafting. Despite its promising results, caution must be exercised when using this technique, particularly in facial cosmetic surgery, to avoid any potential complications. This study aims to conduct a comprehensive literature review on the application and effects of nanofat in regenerative medicine and facial cosmetic surgery.

## 2. Materials and Methods

This review was conducted by searching the PubMed, MEDLINE, and Embase databases on 30 March 2023, supplemented by the authors’ expertise and knowledge. The search terms used were ‘nanofat’ and ‘stromal vascular tissue nanofat’. Only articles published within the past six years were considered, and exclusion criteria included studies without full-text access and those unrelated to facial treatments. To ensure consistency, all authors reviewed and discussed the same papers, and a screening and data extraction manual was compiled prior to the review. The reviewers evaluated the titles, abstracts, and full texts of all papers to determine their relevance, and a data-graphing form was created collaboratively to present the selected articles as a flowchart.

## 3. Results

**Figure 2:**
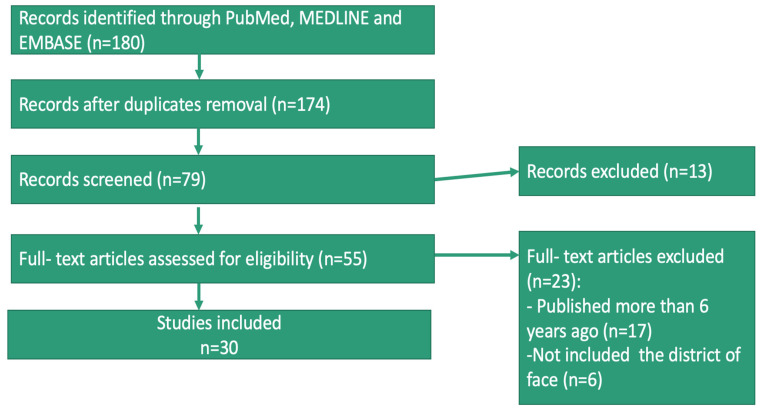
Flowchart.

A total of 180 studies were screened and assessed for eligibility 30 articles met the criteria for inclusion. (Table 1 and Figure 2). 

All data obtained from the 30 included articles, as well as additional relevant papers [1,2,3,4,5,6,7,8,9,10,11,12,13,14,15,16,17,18,19,20,21,22,23,24,25,26,27,28,29,30,31,32,33,34,35,36,37,38,39,40,41,42,43,44,45,46,47,48,49,50,51,52,53,54,55,56,57,58,59,60,61,62,63,64,65,66,67,68,69], were analyzed and are presented in the following paragraphs.

**Table 1:** Studies included in the review.

Authors	Title	Journal	Year of Publication	Main Topics
Batel F.S. et al. [22]	Traitement des ridules de la lèvre supérieure par graisse émulsifiée ou «Nanofat»: étude biologique et clinique à propos de 4 cas	*Annales de chirurgie plastique esthétique*	2018	Evaluate the biological composition of emulsified fat and its clinical effectiveness in the treatment of peri-oral wrinkles in 4 patients aged 50 to 59 years.
Nosheen S. et al. [10]	Unfiltered Nanofat Injections Rejuvenate Postburn Scars of Face	*Annals of Plast. Surg.*	2019	Effect of unfiltered nanofat injection on the quality of post-burn facial scars, with a final 6-month follow-up and evaluation of scars by both the patient and the surgeon using the POSAS scale.
Nabil Fakih-Gomez et al. [23]	Nanofat in Facial Rejuvenation: Step-by-Step Procedure, Patient Evaluation and Recovery Process	*The American J. of* *Cosmetic Surg.*	2021	Intradermal injection of nanofat has been used for skin rejuvenation, texture improvement, and scar treatment. The patients evaluated the treatment using a questionnaire after six months.
Hui Zheng et al. [24]	Conventional Nanofat and SVF/ADSC-Concentrated Nanofat: A Comparative Study on Improving Photoaging of Nude Mice Skin	*Aesthet. Surg J.*	2019	The hypothesis suggests that centrifugation can be employed to obtain SVF/ADSC-concentrated nanofats, which are believed to outperform conventional nanofats in addressing skin photoaging.
Yang Z. et al. [25]	Comparison of Microfat, Nanofat and Extracellular Matrix/Stromal Vascular Fraction Gel for Skin Rejuvenation: Basic Animal Research	*Aesthet. Surg. J.*	2021	The effects of treatment with microfat, nanofat, and SVF-gel on UV-induced skin damage in nude mice were evaluated; the results were formulated by studying the skin using scanning electron microscopy.
van Dongen JA et al. [26]	The Effects of Facial Lipografting on Skin Quality: A Systematic Review.	*Plast. Reconstr. Surg.*	2019	The effectiveness of autologous lipografting in improving skin quality was evaluated, considering a total of nine studies. The review examined various outcomes, including skin texture, color, and elasticity, as well as histologic findings and the incidence of complications.
Uyulmaz S et al. [27]	Nanofat Grafting for Scar Treatment and Skin Quality Improvement.	*Aesthet. Surg. J.*	2018	The effects of nanofat grafting on scars, wrinkles, and skin discolorations were analyzed. Fifty-two patients were treated, and standardized pre- and post-treatment photographs were compared and analyzed to assess the general improvement of skin aesthetics.
Huang R et al. [28]	Nanofat Injection for the Treatment of Depressed Facial Scars.	*Aesthetic Plast. Surg.*	2021	A retrospective study was conducted to evaluate the satisfaction of patients who underwent depressed facial scar filling with nanofat. The patients were asked to provide feedback using the FACE-Q scale.
Surowiecka A. et al. [29]	Adipose-Derived Stem Cells for Facial Rejuvenation.	*J Pers Med.*	2022	Evaluation of the recent evidence obtained from the application of adipose-derived stem cells in facial rejuvenation and their microscopic composition.
Atiyeh B. et al. [30]	Nanofat Cell-Mediated Anti-Aging Therapy: Evidence-Based Analysis of Efficacy and an Update of Stem Cell Facelift.	*Aesthetic Plast. Surg.*	2021	Seven articles published after 2013 have been identified to review any emerging evidence regarding the clinical efficacy of fat grafting as a natural filler.
Bhooshan LS et al. [31]	Autologous emulsified fat injection for rejuvenation of scars: A prospective observational study	*Indian J. Plast. Surg.*	2018	A total of 34 patients were included in this study, which aimed to determine the aesthetic outcome of autologous emulsified nanofat injection in scars. The assessment was conducted using a standardized and validated Patient Observer Scar Assessment Scale (POSAS) and photographs.
Trotzier C et al. [32]	Fat Graft Retention: Adipose Tissue, Adipose-Derived Stem Cells, and Aging.	*Plast. Reconstr. Surg.*	2023	In this review, the authors aim to analyze the effect of aging on adipose tissue components, particularly adipose-derived stem cells (ASCs), that could lead to a decrease in skin regeneration and fat graft retention. Additionally, they seek to examine the parameters involved in graft survival.
Annie Suh et al. [33]	Adipose-derived cellular and cell-derived regenerative therapies in dermatology and aesthetic rejuvenation.	*Ageing Research Reviews*	2019	Aesthetic applications, including hair growth, scar reduction, skin ischemia-reperfusion recovery, and facial rejuvenation, are reviewed. The discussions focus on the conclusions derived from the studies examined.
Madhan J. et al. [3]	Nanofat: A therapeutic paradigm in regenerative medicine.	*World J. Stem Cells*	2021	Literature review on the role of nanofat in modern medicine.
Rihani J. [18]	Microfat and Nanofat: When and Where These Treatments Work.	*Facial Plast. Surg. Clin. North Am.*	2019	The basic principles of microfat and nanofat are explored, including their utilization for enhancing skin texture and achieving structural volume, alongside a detailed description of the injection procedure.
Cohen SR. et al. [12]	Regenerative cells for facial surgery: biofilling and bio contouring.	*Aesthet. Surg. J.*	2017	Fat grafting is now being used for both filling and regeneration.
Gaur M et al. [5]	Mesenchymal stem cells from adipose tissue in clinical applications for dermatological indications and skin aging.	*Int. J. Mol. Sci.*	2017	The identification of the mechanisms by which ADSCs accomplish dermatological rejuvenation and wound healing has great potential to identify novel targets for the treatment of disorders and to combat aging.
Menkes S. et al. [34]	Subcutaneous Injections of Nanofat Adipose-derived Stem Cell Grafting in Facial Rejuvenation.	*Plast. Reconstr. Surg. Glob. Open*	2020	The use of subcutaneous nanofat injections for facial rejuvenation demonstrates promising results, effectively enhancing the skin’s appearance by altering the dermal pattern.
Wei H et al. [35]	Nanofat-derived stem cells with platelet-rich fibrin improve facial contour remodeling and skin rejuvenation after autologous structural fat transplantation.	*Oncotarget*	2017	Transplants that combine newly isolated nanofat, which has a rich stromal vascular fraction (SVF), with PRF and autologous structural fat granules may therefore be a safe, highly effective, and long-lasting method for remodeling facial contours and rejuvenating the skin.
Tonnard P. et al. [13]	Fat Grafting for Facial Rejuvenation with Nanofat Grafts.	*Clin. Plastic Surg.*	2020	Nanofat is a highly concentrated solution of progenitor cells without viable adipocytes. Nanofat grafting creates striking skin quality improvement.
Girard P. [47]	Modified nanofat grafting: Stromal vascular fraction simple and efficient mechanical isolation technique and perspectives in clinical recellularization applications	*Frontiers in bioengineering and biotechnology*	2022	The study analyzed three groups of SVF obtained by 20, 30, and 40 inter-syringe passages. The control group was an SVF obtained by enzymatic digestion. They studied their cell composition by flow cytometry, observed their architecture by confocal microscopy, and observed immunomodulatory properties of the ASCs from each of the SVFs by measuring inflammatory markers of macrophages obtained by an ASC monocyte co-culture.
Qui H. et al. [48]	The Effect of Different Diameters of Fat Converters on Adipose Tissue and Its Cellular Components: Selection for Preparation of Nanofat	*Aesthetic Surgery Journal*	2021	The 3-dimensional finite element method was employed to simulate the process of mechanical emulsification of fat and to research the stress with five different converters (3.76 mm, 2.00 mm, 1.20 mm, 1.00 mm, 0.80 mm). An assessment of the morphology of emulsified fat was conducted. Isolated stromal vascular fraction (SVF) was analyzed for cellular components, number, and viability through flowcytometry and live/dead staining. Adipocytic and angiogenic differentiation assay allowed the assessment of the differentiation capacity of the SVF.
Jan N.S. et al. [10]	Unfiltered nanofat injections rejuvenate postburn scars of face	*Annals Plastic Surgery*	2018	The aim of this study was to compare the quality of postburn facial scars before and after injection of unfiltered nanofat.
Ding P. et al. [36]	Research progress on preparation, mechanism, and clinical application of nanofat	*Journal of Burn Care and Research*	2022	Nanofat grafting is a relatively new technology that has gained popularity and is produced by mechanical shuffling and filtration of microfat. Unlike microfat, nanofat particles are too small to provide a notable volumizing effect. Studies have shown that nanofat contains abundant stromal vascular fraction cells and adipose-derived stem cells, which help reconstruct dermal support structures and regenerate healthier, younger-looking skin. Moreover, the fluid consistency of nanofat allows application in lipofilling such as scars, chronic wounds, and facial rejuvenation.
Sanchez-Macedo N. et al. [37]	Protein profiling of mechanically processed lipoaspirates: discovering wound healing and antifibrotic biomarkers in nanofat	*Plastic and Reconstructive Surgery*	2022	Microfat and nanofat samples were isolated from 18 healthy patients. Proteomic profiling was performed through untargeted mass spectrometry proteomics and multiplex antibody arrays.
Sesè B. et al. [38]	Nanofat Cell Aggregates: A Nearly Constitutive Stromal Cell Inoculum for Regenerative Site-Specific Therapies	*Plastic and Reconstructive Surgery*	2019	The authors compared the number of cells recovered from 1 g of lipoaspirate between stromal vascular fraction and nanofat preparations, and subsequently determined the final cell inoculum obtained following their respective protocols.
Gentile P. et al. [39]	Comparing different nanofat procedures on scars: role of the stromal vascular fraction and its clinical implications	*Regenerative Medicine*	2017	Three different modified nanofat grafting procedures (supercharged-, evo- and centrifuged-modified nanofat) were compared with the classic nanofat method, and histological analysis was performed to assess skin regeneration. Residual nanofat samples were analyzed to determine SVF immunophenotype and yield from each procedure.
Daumas A. et al. [40]	Fat grafting for treatment of facial scleroderma	*Clinical Plastic Surgery*	2020	Fat grafting in scleroderma patients likely improves skin manifestations by recreating fullness, correcting contour deformities, and improving skin quality. The injected fat provides a mixture of cells that influences the recipient site, resulting in improved outcomes.
Bertheumil N. et al. [41]	Mechanically isolated stromal vascular fraction by nanofat emulsification techniques	*Plastic and Reconstructive Surgery*	2017	ASCs isolated by nanofat technique show immunosuppressive properties by decreasing the proliferation of human T cells, by the secretion of paracrine factors, and favoring angiogenesis.
Verpaele et al. [15]	Nanofat needling: a novel method for uniform delivery of adipose derived stromal vascular fraction into the skin	*Plastic and Reconstructive Surgery*	2019	A novel method for delivering nanofat into the skin is microneedling. This technique combines the regenerative capacities of microneedling with those of nanofat injection.

### 3.1. Concept of Nanofat

In 2013, Tonnard introduced the nanofat technique for skin rejuvenation. The technique involved injecting nanofat grafts into various areas, including breast cleavage (eight cases, 11%), glabellar skin (15 cases, 23%), and perioral skin (38 cases, 58%), as well as to treat dark lower eyelids (2%), scars (4%), and other conditions in a cohort of 67 patients. A 27-gauge needle was used for superficial intradermal and subdermal injection, and injection continued until a yellowish tint appeared. Discoloration typically resolved within a few hours after injection, and clinical outcomes improved progressively over time, peaking between 4 and 6 months after surgery. No significant complications, granulomas, infections, fat cysts, or other adverse effects were reported in this series, although brief erythema lasting 1.5 to 2 days occurred when larger areas, such as the face or décolletage, were injected [2]. Nabil Fakih-Gomez treated 20 female patients with nanofat (mean BMI 27.99 + 1.27, mean age 36.8 years) obtained from the thighs (80%) or lower abdomen. Patients received a single dose of nanofat, and the follow-up was conducted at 6 months. Of the treated patients, 65% reported a general improvement, including increased skin smoothness (100%), reduced wrinkles (40%), decreased pore size (15%), and decreased redness (10%). No significant side effects were reported, although minor complications, such as bruising at the donor site (100%) and pain, were more common in patients who received abdominal donor site injections. Nanofat has also been used to treat non-hypertrophic post-burn scars in a study of 48 patients (28% female, mean age of 22.25 + 5.79 years) [10]. Nanofat 2.0 [14] was used, and after 6 months, scar improvement was assessed using the Patient Observer Scar Assessment Scale (POSAS) score. All score parameters improved significantly (*p* < 0.0001), with the greatest improvement being in skin flexibility due to the regenerative effect and fat placement between the scar tissue and underlying tissue. Studies on nanofat are limited by small sample sizes and non-quantifiable subjective skin results. In the future it would be useful to create a score that allows an objective and tangible definition of the aesthetic result obtained. This measurement system could be used in the future for each fat transfer procedure and could be based on the use of a satisfaction questionnaire as happens after most plastic surgery operations. At the present time one of the most well-known scales for aesthetic facial surgery is FACE-Q.

### 3.2. The Three Phases of Nanofat

**Figure 3:**
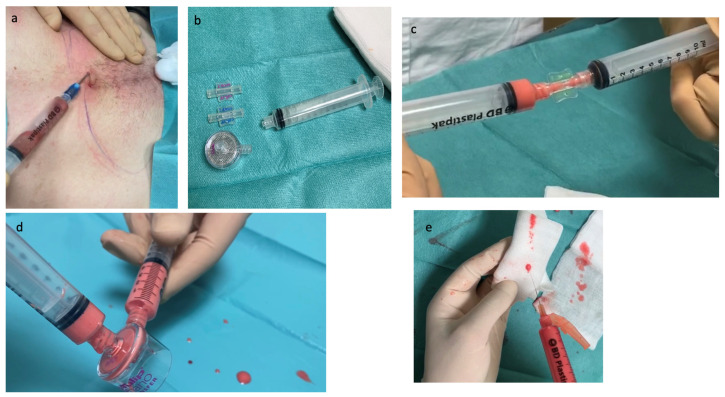
Schematic representation of our method for obtaining nanofat using the Tulip system. Adipose tissue is harvested using a cannula with a 1 mm hole attached to a Luer-lock syringe (**a**). The adipose tissue is then passed back and forth 30 times between two 10 cc syringes connected by a Luer-lock connector, with diameters of 2.4 mm and 1.4 mm, respectively (**b**,**c**). Finally, a single pass is made through a nanofilter (**d**). The resulting emulsion can be infiltrated using 30-gauge needles (**e**).

The most common method for obtaining nanofat involves shuffling lipoaspirate between syringes and filtering it to produce the nanofat product. The process of obtaining nanofat can be divided into three phases. The first phase is the fat harvesting procedure, which involves collecting fat from the lateral side of the thigh (preferably in females) (micro-nanofat), the medial side of the thigh, and the abdomen (the most reliable collection site in males) [18]. The collection site is treated by infiltrating a solution of 1% lidocaine and 1:1,000,000 adrenaline. The fat is collected using a liposuction cannula with 1 mm side ports, taking approximately 40–50 mL of aspirate. The second phase involves the emulsification of the fat, which is achieved by shifting the fat between two 10-cc syringes connected to each other (Figure 3). Once the emulsification and filtering phase is complete, the resulting product is a translucent liquid that is rich in high-quality mesenchymal stem cells but devoid of any viable adipocytes [2]. The final phase involves the injection of the nanofat product into the desired areas of the patient.

### 3.3. How Does It Work?

Adipocytes represent only 25% of the total number of cells in fat tissue, despite accounting for 80% to 90% of its volume [6]. The remaining 75% of the sample, known as the stromal vascular fraction (SVF), is rich in adipose-derived stem cells (ADSCs), endothelial cells, granulocytes, monocytes, macrophages, and lymphocytes. Recent studies suggest that the many cell types in the SVF may work together to create a microenvironment that promotes the regenerative capacity of both stem cells and tissues [5]. Mechanical shear stress applied to fat during nanofat production has been shown to activate signaling pathways that enhance the ability of multipotent and pluripotent stem cells to regenerate [7]. Reports indicate that these intricate intercellular crosstalk events can lead to increased collagen deposition, improved skin suppleness, development of new blood vessels, tissue remodeling, dermal thickening, and downregulation of melanogenic activity [1,8,9,10,11]. Nanofat also appears to reduce inflammation and stimulate angiogenesis [52,53]. The positive effects of ASCs on wound healing are linked to their ability to promote vascular regeneration. ASCs have the capacity to differentiate into endothelial vascular cells, and when co-cultured with endothelial cells they can promote the formation of a vascular network. In comparison to bone-marrow-derived stromal cells, ASC co-cultures develop more junctions and higher network density, indicating their effectiveness in promoting vascular stability. ASCs exhibit features of pericytes and can enhance neovascularization through expression of various growth factors, including VEGFA and insulin-like growth factor-1. The paracrine function of ASCs plays a significant role in their regenerative effects, and their secretome contains a wide variety of factors, including leptin, VEGF, HGF, b-FGF, TGF-β, IL-8, PDGF, PlGF, and SDF-1, which are involved in different stages of angiogenesis. ASCs can form capillary-like tubes, increase endothelial cell growth, and reduce endothelial cell apoptosis through secretion of VEGF, HGF, and TGF-β. The expression of FGF and VEGF can stimulate ASC proliferation, migration, attachment, and endothelial differentiation, and can have a co-stimulatory effect on ASC endotheliogenesis [26,39,40,41,56,57,58,59,60,61,62,63,64,65,66,67,68,69,70,71,72,73,74,75,76]. 

Nanofat stem cells (NSCs) possess strong immunomodulatory effects on both the innate and adaptive immune systems. As shown by Tonnard, the nanofat technique preserves stem cells and shows a marked proliferation capability [2]. They can partially suppress the proliferation of lymphocytes and inhibit the proliferation and differentiation of B-lymphocytes into plasmocytic cells. Treatment with stromal vascular fraction (SVF) cells or ASCs greatly reduces the activities of T-helper 1 and T-helper 17 cells, along with their associated proinflammatory cytokines. The secretome of ASCs is also important for their immunomodulatory and angiogenic properties. However, the characteristics of ASCs may vary depending on patient age, sex, body mass index (BMI), or metabolic state. For example, ASCs derived from patients with type 2 diabetes have been shown to exhibit increased expression of inflammatory markers and reduced immunosuppressive activities. This type of therapy shows promise for the treatment of autoimmune diseases as well [54]. NSCs can modify the behavior of surrounding cells and remodel the extracellular matrix (ECM) in the dermis. ASCs promote the proliferation and migration of dermal fibroblasts and epidermal keratinocytes, both through direct cell-to-cell contact and through the secretion of factors that activate these cells in a paracrine manner. Additionally, ASCs enhance the secretion of ECM proteins, such as collagens and fibronectin, and regulate the synthesis of collagen and matrix metalloproteinases (MMPs) and their inhibitors. ASCs can modulate the homeostasis of MMPs and their endogenous inhibitors, leading to improved collagen organization and decreased expression of α-smooth muscle actin, which are markers of dermal fibrosis improvement. Moreover, ASCs can inhibit the expression of profibrotic factors such as transforming growth factor (TGF)-β1 or IL-6 and increase the expression of the antifibrotic factor TGF-β3. The antifibrotic effect of ASCs is also mediated by their paracrine activity through the secretion of basic fibroblast growth factor (b-FGF), hepatocyte growth factor (HGF), and interleukin-10 (IL-10), which decrease TGF-β1 expression, prevent fibroblast-to-myofibroblast differentiation, and induce myofibroblast apoptosis. This explains the potential benefits of using nanofat as regenerative approach for anti-aging strategies. Nanofat injection is seen as a shift towards a more proactive prevention and maintenance approach, and not only to slow down the aging of facial skin,. It is compared to taking care of a house by periodically replacing carpet or applying more paint to prevent the need for a total renovation. Additionally, the passage mentions injectable tissue replacement and regeneration (ITR) as an innovative approach that combines anatomical fat replacement with regenerative ingredients tailored to the specific needs of the patient. This approach utilizes different types of fat grafting, such as millifat, microfat and nanofat, to replicate the characteristics of fat cells lost with aging and improve the condition of aging skin.

#### How Standardized and Automatized Is the Technique?

Recently Lombardo J.A. et al. designed and fabricated an automatic device that performs emulsification and micronization (EMD) and a filtration device (FD) for lipoaspirate, replacing in this way the manual nanofat procedures. The EMD was designed to process lipoaspirate with greater control. This could lead to a new type of human lipoaspirate samples in terms of stem cell enrichment and numbers compared to traditional methods. Moreover, it significantly enhances mesenchymal stem cells (MSCs) both in terms of cell numbers and relative concentration of epithelial progenitor cells (EPCs) [43].

### 3.4. Nanofat Processing Technique

Currently, the most used nanofat processing technique is Tulip NanoTransfer (Tulip Medical, San Diego, CA, USA). However, it should be noted that there are studies experimenting with other processing techniques in order to modify and improve the cellular content, which is responsible for the biological action. In this study, the authors describe a new processing method [17] that can preserve more matrix, potentially leading to a higher regenerative effect. Avoiding the final filtration step and squeezing the emulsified adipose tissue through a nylon cloth, [14] obtained a new product called Nanofat 2.0, which showed a high quantity of stem cells and a marked proliferative capacity. Another proposed technique combines mechanical emulsification with an enzymatic disintegration step, resulting in the creation of Vivo nanofat [4]. By the Tulip technique, the collected fat is rinsed and filtered through a sterile nylon cloth with a pore size of 0.5 mm mounted on a sterile container. It is mechanically emulsified by a technique involving moving the fat between two 10 cc syringes connected by a female–female Luer-lock connector. According to the Tonnard technique [2], about 30 passes are performed to transform the fat into a white emulsion that requires further filtration, which can be achieved by passing it through a sterile nylon cloth or other appropriate filters to remove any residual connective tissue and allow the effluent obtained to pass through a 27–30 Gauge needle (Figure 2). There is another technique pioneered by Verpaele et al. that could also optimize skin regeneration. The difference is that the injection is performed using a microneedling tool to deliver nanofat. For large areas, such as the complete face, neck, and décolletage, this method is particularly successful. Twenty 1.5 mm needles are included in the device, and the microchannels are formed by repeatedly tapping the instrument. The punctate bleeding obtained indicates that the papillary dermis has been reached [15]. Through the activation of many growth factors, microneedling promotes the induction of percutaneous collagen [16]. It may be possible to maximize outcomes by combining nanofat’s capacity for regeneration with the additional stimulation of collagen synthesis and scarless repair offered by microneedling [12].

### 3.5. What Kind of Treatment Is It Possible to Combine with Nanofat?

In order to provide a shorter-term enhancement of skin quality, the authors used a novel delivery system which combined microbotox, skin booster HA, and vitamin C. Indeed, a faster change in the appearance of the skin was seen when compared with nanofat delivery only [13]. It also used microfat which is made up of intact and vital adipocytes with their cellular environment. Microfat acts as a traditional fat graft, so adipocytes are incorporated into the injection site, providing volume, mechanical and structural support while the nanofat acts as a regenerative agent [2].

### 3.6. Nanofat as a Treatment for Facial Rejuvenation

The adipose tissues in the face are divided into different sections by fibrous connections called ligaments and septa. These ligaments and septa, with unique characteristics of vascularity, thickness, and consistency, play an important role in the organization of facial tissues. Facial ligaments are solid fibrous structures that originate from the periosteum (the connective tissue covering the bones) or the parotid-masseteric fascia. They extend vertically towards the skin, adhering to the dermis and providing a stable anchorage between the skin and deeper tissues, such as the superficial musculoaponeurotic system (SMAS). On the other hand, fibrous septa are extensions of ligaments into the subcutaneous adipose tissue. These septa create distinct compartments, separating and delimiting different sections of adipose tissue in the face. Superficial adipose compartments are located above the facial mimic muscles and do not cover the transition area between the malar region, the infero-lateral eye angle, and the zygomatic arch. They are separated by fibrous septa. Deep adipose compartments, on the other hand, are located below the SMAS, just above the orbital periosteum and the maxillary and zygomatic bones. These compartments are deeper than the mimic muscles and are separated by fibrous ligaments.

In the malar region (cheek), various ligaments can be found, such as the zygomatic ligament that delimits the region superiorly, and the masseteric ligament that vertically divides the area into two parts. This area is primarily treated through deep adipose injection to restore the tone of the ligaments and promote angiogenesis in the soft tissues and bone surface [12]. Around the orbit (eye), we find the orbicular ligament that surrounds the eye region almost completely, and the mandibular ligament, which extends to the areas where the platysma muscle inserts into the bones. The presence of these ligaments and fibrous septa in the subcutaneous adipose tissue helps connect the SMAS to the dermis, allowing each layer of tissue to move independently during facial expressions. The forehead, divided into three regions (central, left temporal, and right temporal), determines the position of the eyebrows and glabellar projection. Injections in this region can be beneficial for reducing sun damage and promoting lipomodelling. The temporal areas can be treated with injections above the superficial layer of the deep temporal fascia, which contains veins and the temporoparietal fascia, achieving a volumizing effect and reducing the visibility of veins [12].

Facial rejuvenation with nanofat has become an increasingly popular technique due to its ability to provide natural and long-lasting results with minimal downtime. Nanofat is a form of fat grafting that involves harvesting small amounts of fat from the patient and processing it to obtain a liquid or semi-liquid form that can be injected into the face. Nanofat has been shown to improve skin texture and tone, stimulate collagen production, and promote neovascularization. The procedure is minimally invasive, with most patients experiencing only mild swelling and bruising that typically resolves within a few days. In 2013, a review entitled “Stem cell facelift: between reality and fiction” was published [55]. At that time, there was no clinical evidence supporting this procedure. However, since then, the enthusiasm around the promises of stem cell regeneration has decreased, but interest in this area of research continues to grow. In that same year, the technique of nanofat grafting was described for the first time [2]. Nanofat has been found to be effective in producing a face-lifting effect, as described by Sophie Menkes et al. [34]. In this study, 50 patients (two men and 48 women aged 35–65 years) were enrolled and treated with subcutaneous injections of 18 mL nanofat-PRP to evaluate its regenerative and face-lifting effects. Results were evident between weeks two and four after treatment, with patients reporting improvements in texture, elasticity, hydration, and radiance of facial skin. These effects are attributed to the modification of the dermal pattern, as evidenced by the biopsies performed. Skin biopsies of the post-treatment zone showed an increase in collagen and elastic fibers in the superficial dermis. Moreover, no major side effects were reported, except for edema, which disappeared a few days after therapy, and pain at the donor site. Most patients reported not needing to apply makeup and confirmed improvements in texture, glow, firmness, and fine wrinkles. More than 80% of patients exhibited a large improvement in facial shape. In the field, a long-term effect has been demonstrated how nanofat induces collagen and elastin synthesis by the power of stem cells without changes in pigmentation of the skin over a 1-year follow-up [70]. The clinical results in term of rejuvenation were apparent between two and four weeks after injection, and improvements were continuously observed to be stable over time until six months postoperatively. Histologically, besides the increase in collagen deposition and elastic fibers, a large percentage of patients have an increase in epidermal thickness, vascular density and normal rete ridges at 1 year after treatment [15,34]. Nevertheless, stable long-term results remain the greatest challenge in aesthetic rejuvenation. At this time, no contraindication to repeat this procedure has been described in the literature. In conclusion, nanofat represents a possible alternative to traditional face-lifting methods, appearing to be a very effective and safe approach for modifying the dermal pattern.

### 3.7. Long Term Outcomes and Patient Satisfaction

**Figure 4:**
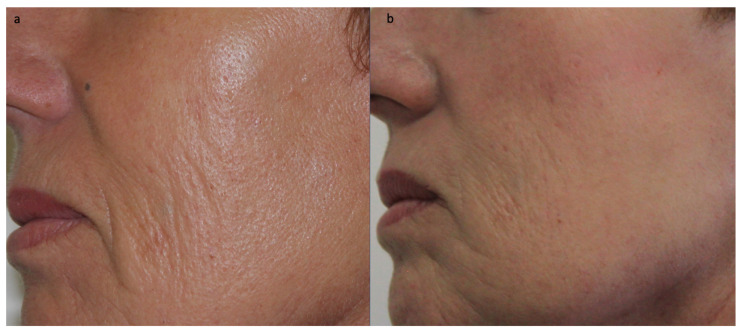
A 53-year-old patient approached us to correct facial fine lines (**a**). An amount of 5 cc of nanofat (obtained from adipose tissue harvested from the hips under local anesthesia) were injected. No anesthesia was used for the facial injections. After one year of treatment, an improvement in wrinkles can be observed (**b**), and the patient was highly satisfied.

Physicians’ evaluations have confirmed that patient satisfaction remains stable after nanofat treatment (Figure 4) [28]. Some patients have reported continuing improvement in the injection areas even three years after the fat grafting procedure [49]. Better survival rates of the injected fat can be partially attributed to angiogenesis, as it is believed that the reduction in microvasculature and nutrients in aging skin are responsible for the changes observed in aging skin [40,46]. Studies have shown a very low rate of injection-site complications and established the safety of the procedure. In fact, no significant complications have been reported except for temporary erythema, which appeared at the injection site with varying degrees and lasted for 2–3 weeks in the majority of patients. The risk of complications can be minimized by using fine injection techniques and small injection volumes [28]. Unlike macrofat and microfat, nanofat has not been associated with infections, granulomas, or fat cysts [50,51].

In the Table 2 below we summarize strengths, opportunities, weaknesses and threats of this fat transfer technique.

### 3.8. Are There Any Possible Alternatives to Nanofat?

Numerous techniques and concepts have been proposed in the field of fat grafting to improve the quality and retention of facial fat grafting. These techniques can be broadly divided into two categories: those that incorporate extra components such as platelet concentrates and stem cells, and those that use fat preparations such as stromal vascular fractions (SVFs) and nanofat. Stem-cell-based techniques utilize adipose-derived stem cells (ADSCs) and bone-marrow-derived stem cells (BMSCs), which have direct differentiation ability and paracrine capability, and can be used in slim patients to provide abundant resources. Fat-preparation-based techniques, such as nanofat, SVFs, and SVF-gel, have shown high effectiveness in both volumization and rejuvenation. Platelet-concentrate-based techniques, such as PRP, PRF, and CFG, have also been investigated, with PRP being the only one approved by the Food and Drug Administration [39,40,41,46,47,48,49,50,51,52,53,54,55,56,57,58,59,60,61,62,63,64,65,66,67,68,69]. Several studies have explored the use of nanofat for the treatment of various skin changes. In one study, perioral wrinkles in four patients were treated with high patient satisfaction after four months of intradermal injection [22]. Nanofat has also been used in combination with other substances, such as platelet-rich fibrin, to treat skin aging in 103 patients, with excellent results in improving skin texture and patient satisfaction [35]. Another study showed an additional effect of nanofat, which is effected by ADSCs that can reduce melanin synthesis by acting on tyrosinase. Nineteen patients were treated to reduce skin pigmentation of the lower eyelid, and a marked improvement in skin discoloration was recorded four months after treatment [36]. While there are other techniques that have been explored, the effectiveness and safety of nanofat make it a promising option for facial rejuvenation especially for patients seeking a minimally invasive procedure.

## 4. Discussion

The appearance of a person’s skin is indicative of their lifestyle habits and can be influenced by two major factors: health and youth. Patients value skin beauty as it contributes to their self-confidence and success. The nanofat grafting technique is designed to enhance lipomodelling, neocollagenesis, vascularization, and skin physiology. It utilizes ASCs, which not only have a multipotent nature but also secrete various stimulatory factors such as cytokines, chemotactic factors, growth factors, and their inhibitors. These factors perform complex functions and play a significant role in skin rejuvenation and repair of various functions such as melanocyte action. The interleukin-6-mediated mechanism of ASCs aids in inhibiting melanocyte proliferation and melanin synthesis, resulting in positive outcomes in the treatment of dark coloration of lower eyelid skin [1,2,3,4,5,6,7,8,9,10,11,12,13,14,15,16,17,18,19,20,21,22,23,24,25,26,27,28,29,30,31,32,33,34,35,36,37,38,42,43,44,45]. In a prior research study, Tonnard et al. assessed stem cell quality by quantifying cells from the SVF and its CD34+ subfraction, culturing the cells in standard and adipogenic media. They found a higher number of ASCs, which are associated with increased regenerative capacity, potentially contributing to the observed lifting effect [2], than in microfat samples. Nanofat processing involves more than just breaking down adipocytes and can lead to an upregulation of markers that induce regenerative capacity. Injection of the product subdermally in multiple areas of the face led to improvements in facial appearance without a change in volume. Hyaluronic acid, lipofilling, PRP, mesotherapy with peptides and vitamins, lasers, ultrasound, and radiofrequency have also been used to improve skin quality, but these modalities only have a moderate impact and can cause complications. Fillers have been reported to cause lumps, bruising, and irregularities.

The lower third of the aging face, particularly the lips and perioral area, should receive more attention in facial rejuvenation procedures. Nanofat injections in the subcutaneous layer could expand the use of this technique to sensitive areas, such as from the zygomatic arch to the temporal area, without adverse effects. The nanofat procedure takes 30 to 45 min for infiltration, harvesting, and emulsification, and PRP is centrifuged before fat harvesting to reduce the procedure time if the procedures are associated. The injection time with a cannula or a 30-gauge needle is similar to that of filler treatments. Swelling may occur for 2–4 days, along with bruising and pain at the donor site for a week, but no delayed reactions have been reported over 5 years. Cannula injection is preferred to avoid vascular complications, or alternatively, small aliquots may be injected under low pressure using a needle.

The nanofat technique has some disadvantages, such as the need for a surgical procedure and discomfort associated with extracting fat from the donor site. A challenge to improve the accuracy and repeatability of this technique is to count the stem cells of the stromal vascular fraction and adipocytes in the fat and after obtaining the nanofat for each sample. Indeed, the success of the procedure also depends on various factors such as careful monitoring of each step of the process including the choice of donor site, harvesting, processing, and injection techniques. Disinfection is a crucial aspect of the process, but local antiseptic treatment can affect the viability of adipose-derived stem cells (ASCs). Some antiseptics such as povidone iodine and polyhexanide can decrease ASC viability and reduce the expression of stem cell markers. However, in the study, povidone iodine was used, and the area was carefully washed with saline solution to avoid any introduction of antiseptic inside the tissues. In previous studies, it was found that the abdominal wall is the best site for harvesting fat due to the high number of stromal vascular fraction (SVF) cells and growth factors present. However, there was no significant difference in the count of adipose-derived stem cells (ASCs) among the harvest sites. It is also important to limit the amount of anesthesia used during the procedure to protect the stem cells, as lidocaine has been found to be toxic. PRP alone is effective on skin rejuvenation, but a minimum of three sessions are still necessary to obtain results. In a previous work of our team on PRP as a potentialisator of cell therapy in the field of plastic surgery, 20% PRP associated to 80% of nanofat showed the best efficiency [63]. In this review, we identified 30 primary studies on nanofat, with a particular focus on its use in facial applications. Despite the potential benefits, there are only a few studies on facial applications of nanofat, and it is still underutilized in this area. However, recent advancements in the field have demonstrated that nanofat is one of the best ways to improve skin texture and facial shaping. As such, we believe that nanofat and regenerative medicine are important therapeutic resources for treating facial conditions. We aim to share our most recent knowledge to achieve higher-level outcomes and minimize complications. Further studies are needed to improve patient selection, such as using the Fitzpatrick Score and other scales such as the Glogau Scale for wrinkles. The wide field of application of nanofat is a recent achievement in the field of regenerative medicine, and we believe it has great potential in improving the quality of life for patients.

## 5. Conclusions

The available methods for improving the quality of facial skin are effective, but subcutaneous nanofat injections have emerged as a highly efficient technique for facial rejuvenation. This is due to their ability to modify dermis patterns. Nanofat grafting is a regenerative technique that involves the use of autologous procedures and can be performed on an outpatient basis. Clinical trials have demonstrated the safety of this approach, and nanofat integrates seamlessly with host tissues without causing significant side effects. The traditional approach to facial aging is undergoing significant changes. Innovative therapies targeting different levels of facial tissues will be developed, allowing for optimal results. Regenerative medicine will play a crucial role, not only improving appearance but also regenerating tissues, offering non-invasive solutions. For example, volume augmentation using biomaterials such as autologous fat can stimulate tissue regeneration and potentially slow down cellular aging by addressing early anatomical, cellular, and histological changes. Further research is needed to study these new techniques as they represent a new frontier not only in aesthetic medicine but also in lipofilling across various medical fields.

## Figures and Tables

**Table 2:** SWOT analysis of nanofat.

Strengths	Opportunities	Weaknesses	Threats
Minimally invasive	Use of this technique as a bridge to surgery in severely burned patients	Few studies dills about long term effects	New techniques of fat transfer are being studied
Patient’s satisfaction	Combinable with other face rejuvenation procedures	Not yet a standardized technique	The high impact of dermal filler in aesthetic field

## Data Availability

Data is unavailable due to privacy or ethical restrictions.
